# Different responses of growth and physiology to warming and reduced precipitation of two co-existing seedlings in a temperate secondary forest

**DOI:** 10.3389/fpls.2022.946141

**Published:** 2022-10-14

**Authors:** Junfeng Yuan, Qiaoling Yan, Jing Wang, Jin Xie, Rong Li

**Affiliations:** ^1^ Qingyuan Forest Chinese Ecosystem Research Network (CERN), National Observation and Research Station, Shenyang, China; ^2^ Chinese Academy of the Sciences (CAS) Key Laboratory of Forest Ecology and Management, Institute of Applied Ecology, Shenyang, China; ^3^ University of Chinese Academy of Sciences, Beijing, China; ^4^ School of Life Sciences, Zhengzhou University, Zhengzhou, China; ^5^ Key Laboratory of Agricultural Water Resources, Hebei Key Laboratory of Soil Ecology, Center for Agricultural Resources Research, Institute of Genetic and Developmental Biology, Chinese Academy of Sciences, Shijiazhuang, China

**Keywords:** warming, drought-intolerance species, foliar N content, root biomass, specific area weight

## Abstract

Warming and precipitation reduction have been concurrent throughout this century in most temperate regions (e.g., Northeast China) and have increased drought risk to the growth, migration, or mortality of tree seedlings. Coexisting tree species with different functional traits in temperate forests may have inconsistent responses to both warming and decreased precipitation, which could result in a species distribution shift and change in community dynamics. Unfortunately, little is known about the growth and physiological responses of coexisting species to the changes in these two meteorological elements. We selected two coexisting species in a temperate secondary forest of Northeast China: *Quercus mongolica* Fischer ex Ledebour (drought-tolerant species) and *Fraxinus mandschurica* Rupr. (drought-intolerant species), and performed an experiment under strictly controlled conditions simulating the predicted warming (+2°C, +4°C) and precipitation reduction (-30%) compared with current conditions and analyzed the growth and physiology of seedlings. The results showed that compared with the control, warming (including +2°C and +4°C) increased the specific area weight and total biomass of *F. mandschurica* seedlings. These were caused by the increases in foliar N content, the activity of the PSII reaction center, and chlorophyll content. A 2°C increase in temperature and reduced precipitation enhanced root biomass of *Q. mongolica*, resulting from root length increase. To absorb water in drier soil, seedlings of both species had more negative water potential under the interaction between +4°C and precipitation reduction. Our results demonstrate that drought-tolerant species such as *Q. mongolica* will adapt to the future drier conditions with the co-occurrence of warming and precipitation reduction, while drought-intolerant species will accommodate warmer environments.

## Introduction

The natural and anthropogenic activities have largely changed global climatic conditions, which pose a threat to life on earth (Hodson, 2017). Moreover, the global surface temperature has increased during the past decades and it is expected to continue rising by 0.3-4.8°C at the end of this century (IPCC, 2013). Warming and a shift in precipitation patterns have caused and exacerbated regional drought conditions, especially in mid/high-latitude temperate regions (IPCC, 2013; Li et al., 2017). Northeast China is located in the middle and high latitudes of the northern hemisphere and has experienced a much more rapid warming trend than the national and global scales for the last decades (Piao et al., 2010). Furthermore, the precipitation amounts of Northeast China have approximately decreased by 30% and suffered from increased drought risk in recent studies (Gao et al., 2020). Taken together, Northeast China is typical among the areas where warming and drought will be concurrent throughout this century. These climatic alterations may potentially alter the interaction between plants and their environment, and thus alter forest structure and function because of certain species migration or mortality. Accordingly, to improve the ability to forecast forest dynamics, there is a compelling need to assess the responses of plants to these combined changes in temperature and precipitation (Hu et al., 2013).

Most previous studies analyzed the effects of climate change on adult trees (Morin et al., 2010) without considering seedlings. However, seedlings are more sensitive to climate variability than adult counterparts due to their limited root system and leaf areas, leading to less carbohydrate accumulation and resource acquisition in seedlings that cannot cope well with abrupt environmental changes (Lloret et al., 2009). Thus, seedling recruitment is considered a critical stage for the successful establishment of tree species. The previous study has indicated that the seedling mortality of dominant tree species in temperate forests increased, and seedling growth was limited under future climates (Yang et al., 2012). The shrink of distribution and even a decrease in community species richness may happen in temperate regions in the future.

A temperature increase and a precipitation decrease are expected to affect individual growth, carbon or biomass accumulation and allocation pattern, and physiological processes, and thus change species survival, establishment, and the composition of ecosystems (Hu et al., 2013; Reich et al., 2018). In general, warming will promote individual growth and photosynthesis as long as the temperature does not exceed the optimum range in alpine ecosystems and polar regions, but it is still uncertain for temperate regions (Milbau et al., 2017; Han et al., 2019). Yun et al. (2016) found warming enhanced the growth of root collar diameter and increased chlorophyll contents of *Pinus densiflora* Sieb. et Zucc. seedlings. A meta-analysis of data from 24 studies showed that the above-ground biomass, growth, and photosynthesis increased with elevated temperatures (Wu et al., 2011). However, decreased precipitation usually suppresses total leaf area to decrease water loss, increases root length and root biomass to enhance water uptake capacity responding to reduced soil moisture, and finally limits the growth of plants (Rodgers et al., 2018). Hence, when considering the combined effects of temperature and water on seedling growth in the future, individual performance might determine the balance of response to warming and precipitation reduction. However, there is little information about the interactive effects of warming and decreased rainfall on seedlings’ growth and physiology.

The impacts of warming and reduced precipitation on the growth and physiology of seedlings differed across changing intensity (Taeger et al., 2015) and among species (Matías et al., 2016). Although the 2 °C temperature increase is used for most works (Xiao et al., 2019; Lyons et al., 2020), it is not representative of many sites, where projected temperature alterations by the end of the current century have been well above 2 °C, e.g., the Tibetan Plateau (Zhao et al., 2022) and northern latitude mountains (Nogués-Bravo et al., 2007). As a consequence, a larger temperature magnitude is needed for the study. The responses of different species to climate change depend on exogenous and endogenous factors, such as habitats and species traits. The habitat can shape the endogenous climate stress tolerance of species, where species from xeric habitats are more adapted to warming and lower water availability than those from humid habitats. For example, *Pinus halepensis* Mill. and *Pinus pinaster* Ait. from drier habitats (lowland) showed a higher survival and better performance in warming and direr habitats than in humid habitats (high-elevation) (Matías et al., 2016). Different functional traits of species (e.g., shade tolerance and drought tolerance) also facilitate trees to accommodate environmental alternations (Richter et al., 2012). This induced the majority of research to focus on the comparison among various provenances and families of species (Kueppers et al., 2017; Zhu et al., 2020). But the consequences of warming and decreased precipitation could be of paramount importance for coexisting species with contrasting traits since inconsistent responses of these coexisting species to climate change could result in a species distribution shift and change in community dynamics (Chmura et al., 2017; Reich et al., 2018). Unfortunately, little is known about the growth and physiological responses of coexisting species to both warming and decreased precipitation.

Secondary forest is the main forest type across the world and Northeast China, accounting for 60% and 72% of forest area, respectively (Zhu et al., 2018). However, compared with the primary stands, there have been many problems in secondary forests, e.g., productivity losses and stability and resilience declines. These problems are radically caused by the poor regeneration capacity of dominant species (Yu et al., 2017). As a consequence, improving their natural regeneration would determine the future destiny of secondary forests. Evaluating seedling performance to climate alterations is vital to enhance regeneration and consequently restore secondary forests. Specifically, projected warming and drought both will induce lower soil moisture. Thus, the traits related to water balance might play a key role in the future performance of seedlings. As precious commercial timber species, *Quercus mongolica* Fischer ex Ledebour and *Fraxinus mandschurica* Rupr are dominant and co-existing in secondary forests of Northeast China. In addition, *Q. mongolica* is mainly distributed in uphill xeric environments with sunny slopes and belongs to a drought-tolerant species (Dai et al., 2020). While *F. mandshurica* belongs to a drought-intolerant species and tends to grow in comparatively humid sites such as river banks and shady slopes (Liu et al., 2015). The response of these coexisting species with contrasting traits to both warming and decreased precipitation is an essential means of predicting the community dynamics of secondary forests.

Here, we selected two-year-old seedlings of *Q. mongolica* and *F. mandschurica*, and conducted a controlled experiment to compare their physiology and growth under simulated current climatic patterns and projected future climatic scenarios. The patterns of warming included three temperature scenarios (i.e., control, +2°C, and +4°C) and two precipitation scenarios (i.e., control and -30%). We hypothesized that (i) warming would alleviate the effects of precipitation reduction on the seedling physiology and growth, and (ii) seedlings of the drought-tolerant species, *Q. mongolica*, are better adapted to future climate change (warming, precipitation reduction, and their co-occurrence) than seedlings of the drought-intolerant species, *F. mandshurica.* We expect that the different responses of coexisting species to warming and reduced precipitation can provide us with information on future community dynamics and restoration by promoting natural regeneration under climate changes in temperate secondary forests.

## Materials and methods

### Experimental setup

Two-year-old seedlings of the two tree species with the same height (12.7 cm and 9.6 cm for *Q. mongolica* and *F. mandshurica*, respectively) and root collar diameter (1.75 cm and 1.62 cm for *Q. mongolica* and *F. mandshurica*, respectively) were collected from Qingyuan Forest CERN, Northeast China (41°51’N, 124°54’E). There are typical temperate broadleaved secondary forests in Qingyuan Forest CERN. The average annual rainfall in the study area ranges from 700 and 850 mm, 80% of which falls from June to August. The mean annual temperature is +4.7°C. The coldest month is January with a mean air temperature of -12.1°C, and the hottest month is July with a mean air temperature of +21.0°C.

Seedlings were collected on 1st June 2020 and then were immediately transferred to tubular PVC pots (7.5 cm in diameter and 40 cm high) filled with soil. The soil in pots was derived from topsoil from secondary forests in Qingyuan Forest CERN to a depth of 0-10 cm, passed through a 1 cm sieve to remove smaller stones and other impurities, and then autoclaved at 121 °C for 2 h.

After planting, all seedlings were thoroughly irrigated and placed into three incubators (MGC-450BP-2L, inner space with 0.7 m length × 0.55 m width × 1.14 m height) that simulated three temperature scenarios (control, +2°C, and +4°C treatments, respectively) with the temperature treatment as a whole plot factor (**Table 1**): (i) ‘control temperature’, representing the mean monthly day and night temperature of the Qingyuan Forest CERN during the growing season from 2005 to 2019; (ii) ‘+2°C treatment’, simulating an increase of 2°C in day and night mean temperature respecting to the temperature at the Qingyuan Forest CERN by 2055; (iii) ‘+4°C treatment’, simulating 4°C increase than control temperature records by the end of the 21st century (Nogués-Bravo et al., 2007). The weekly temperatures (day/night, in °C) of the study area (Qingyuan Forest CERN) during the 16-week experiment were developed for the three temperature scenarios (control, +2°C, and +4°C treatments, respectively) (**Table 1**).

**Table 1:** Weekly temperatures (day/night, in °C) and light time of day or night (day/night, in hours) during the experiment development in different conditions: control, +2 °C, and +4 °C.

Week	Equivalent	Control	+2 °C	+4 °C	Light time
1-4	June	21.9/15.3	23.9/17.3	25.9/19.3	14.5/9.5
5-8	July	24/18.5	26/20.5	28/22.5	14.5/9.5
9-12	August	23.1/18.3	25.1/20.3	27.1/22.3	13/11
13-16	September	18/11.5	20/13.5	22/15.5	12/12

These values were obtained as monthly mean from the meteorological stations of Qingyuan Forest CERN during the 2005-2019 series.

Two precipitation treatments representing current and future precipitation (CP and FP hereafter) respectively were set up in each incubator, and were applied as a subplot factor: (i) ‘current precipitation’, derived from the mean precipitation of Qingyuan County (~20 km away from Qingyuan Forest CERN) during the growing season (June-September) for 1963-2013 series (138.1 mm); (ii) ‘future precipitation’, simulated precipitation reduction by 30% based on the previous level, as projected of Qingyuan County, Northeast China (96.7 mm). Total precipitation amounts were split into 32 irrigation events (twice per week) from the 1st experimental week to the 16th week. Thus, our experiment was designed with three factors (temperature, precipitation, and species), in which temperature included three levels and both other factors included two levels, fully crossed with 15 replicated seedlings per factor combination (2 × 3 × 15 = 90 seedlings).

For the whole experiment, light intensity in each incubator was constantly fixed at a photosynthetic photon flux density of 105 µmol m^2^ s^−1^ for daytime, which represented the light availability for the understory with moderate openness. The light time was in line with the monthly day length which was based on the time of sunrise and sunset per month in Qingyuan Forest CERN (see **Table 1**), rising gradually at dawn and decreasing at dusk for around one extra hour. To avoid any possible chamber effects, all seedlings were rotated through three different incubators every ten days by randomizing each seedling position within each incubator.

Soil moisture (volumetric water content) was recorded twice a week (two days after each watering event) by using the time-domain reflectometer (TDR) (SM200; Delta-T Devices, Cambridge, UK) during the whole experiment. The TDR with three steel rods was vertically inserted at 5 cm of the soil surface in each pot. The soil moisture was measured on the half of seedlings (i.e., N = 8 per species and treatment combination). Temperatures within the incubators were periodically checked with independent temperature devices (iButton, DS1922L) continuously logged to ensure the temperature accuracy of different treatments. To measure temperature, the temperature device was installed in the center of the incubator and logged at 1-hour intervals. The study was performed for 16 weeks from 26 June 2020 to 16 October 2020 at incubators.

### Seedling measurement

Before we transplanted seedlings, an initial measure of growth variables (i.e., root collar diameter (RCD), height, and maximum root length) was conducted for all seedlings. The RCD was recorded at a constant height (1 cm above the soil surface) using a digital caliper, and seedling height was measured as the distance from the root collar to the tip of the top bud. The seedlings were laid on a flat surface and the roots were straightened to measure the maximum root length. The maximum root length was measured from the base of the stem to the tip of the root system. At the end of the experiment, we again measured the final three growth variables of all seedlings.

The leaf areas of nine fresh plants for each treatment were measured by the high-resolution scanner (Founder Z1600, China). The acquired pictures were analyzed by computer image analysis software (Díaz-Barradas et al., 2010). In addition, the maximum photochemical efficiency of photosystem II (Fv/Fm) and the plant water potential (Ψ) of three replicates per treatment were measured before the final harvest of seedlings (Tuna et al., 2008; Zheng et al., 2019). Fv/Fm was recorded with a portable fluorometer (FMS2, Hansatech Instruments, UK) at predawn. A fiber-optic encased in a light-tight chamber was inserted onto the leaf clip, and the healthy and fully expanded leaves were exposed to measured light (0.05 µmol m^−2^ s^−1^). After getting the minimum fluorescence yield (F_0_), the leaves were given a saturating pulse of actinic light (300 mmol m^−2^ s^−1^) for 0.7 s to read a maximum fluorescence yield (Fm). The maximum quantum yield of photosystem II (Fv/Fm) was calculated as Fv/Fm = (Fm −F_0_)/Fm. A fully expanded mature leaf in the upper canopy was randomly selected to measure the water potential (Ψ) by using a pressure bomb (range: 0-15 MPa; PMS, Manofrígido, Lisbon, Portugal). Ψ was recorded on the same day at predawn (03:00-05:00). Then nine intact plants were divided into three parts (i.e., leaves, shoots, and roots), and the harvested roots were carefully washed to remove the remnants of soil. The three parts of the seedlings were oven-dried at 60°C for 72 h to a constant weight, and the dry biomass of each part was recorded.

### Carbon and nitrogen analyses

We randomly collected nine plants and divided them into three groups per treatment combination to analyze carbon and nitrogen concentrations. Dried leaves were ground to powder, passed through a 0.25 mm (60 mesh) sieve, and analyzed for total carbon and nitrogen concentrations. Foliar carbon (C) and nitrogen (N) concentrations were measured by an elemental analyzer (vario MICRO cube; Elementar Analyser Systeme GmbH, Hessen Hanau, Germany). Finally, we calculated the C:N by dividing foliar carbon concentrations by nitrogen concentrations.

### Determination of chlorophyll pigments

Before final harvest, fresh leaves (500 mg) from two seedlings per treatment were cut up into three groups and homogenized with 10 mL acetone (80%), and then chlorophyll was extracted from the homogenized plant materials to avoid light for 1-2 days. The absorbance of the supernatant was read at 645 nm and 663 nm using a spectrophotometer (UA1880, Jinghua Instruments, China). The relative amount of chlorophyll a, chlorophyll b, and the total content of chlorophyll were calculated according to the following equations:


(1) Chlorophyll a (mg/g) = (12.7×A663−2.69×A645)V/W

(2) Chlorophyll b (mg/g) = (12.9×A645−4.68×A663)V/W

(3) Total Chlorophyll (mg/g) = (20.2×A645+8.02×A663)V/W

where A663 and A645 are the absorbances at 645 nm and 663 nm. V is the final volume of chlorophyll extract in 80% acetone. W is the fresh weight of tissue extracted (500 mg) (Arnon, 1949).

The ratio of chlorophyll a and chlorophyll b (chlorophyll a: b) was calculated by chlorophyll a content divided by chlorophyll b content.

### Data analysis

To evaluate the mean periodic increment of RCD (RCD_inc_), height (H_inc_), and maximum root length (R_inc_), we calculated the difference between the final measured value (V_T1_) and initial value (V_T0_) for these three growth variables as follows:


$$RCD_{inc}/\;H_{inc}/\;R_{inc}=\;V_{T1}-\;V_{T0}$$

The specific leaf weight was determined by the leaf area divided by the leaf dry weight. The root-shoot ratio was calculated by root dry weight divided by the total dry weight of the leaf and stem.

The variation in soil moisture among the different treatments was tested by repeated-measures analysis of variances (ANOVA). The differences in increment of height, RCD and root length, total biomass, root-shoot ratio, Ψ, Fv/Fm, total chlorophyll content, chlorophyll a: b, foliar nitrogen content, and C:N were tested by using three-way ANOVAs across the different factors and interactions. Tukey’s *post hoc* tests were used to further examine the variances across treatment levels. The effect of the experimental incubators could not be assumed as a factor in the ANOVAs since plants were rotated within and between incubators during the whole experiment which minimized the compound effect. The assumption of normality of data was evaluated with a normal probability plot and Shapiro-Wilk tests. The homogeneity of variances was assessed using Levene’s test (Kardol et al., 2006; Buters et al., 2019). Non-normal variables were transformed (log, square root, etc.) to meet the statistical requirements when necessary. A *p*-value less than 0.05 was regarded as statistically significant. All the analyzes were performed with R version 4.0.4 (R Core Team, 2021). A car package (Fox and Weisberg, 2011) was used for normality tests and ANOVAs. A Lsmeans package (Lenth, 2016) was used for Tukey’s *post hoc* tests. The results were shown as mean ± S.E. throughout the paper.

## Results

### Soil moisture

Soil moisture was affected by three factors (temperature, precipitation, and species) and their interactions during the experiment. Soil moisture in CP conditions was significantly higher than in FP conditions for both species for all three temperature scenarios (40.8 ± 0.1 vs 34.0 ± 0.1%; F = 1331.8, *P*< 0.0001). Under the two precipitation treatments, the lowest soil moisture was in the +4°C treatment (35.7%) for both species (see **Supplementary** **Figure 1**).

**Figure 1:**
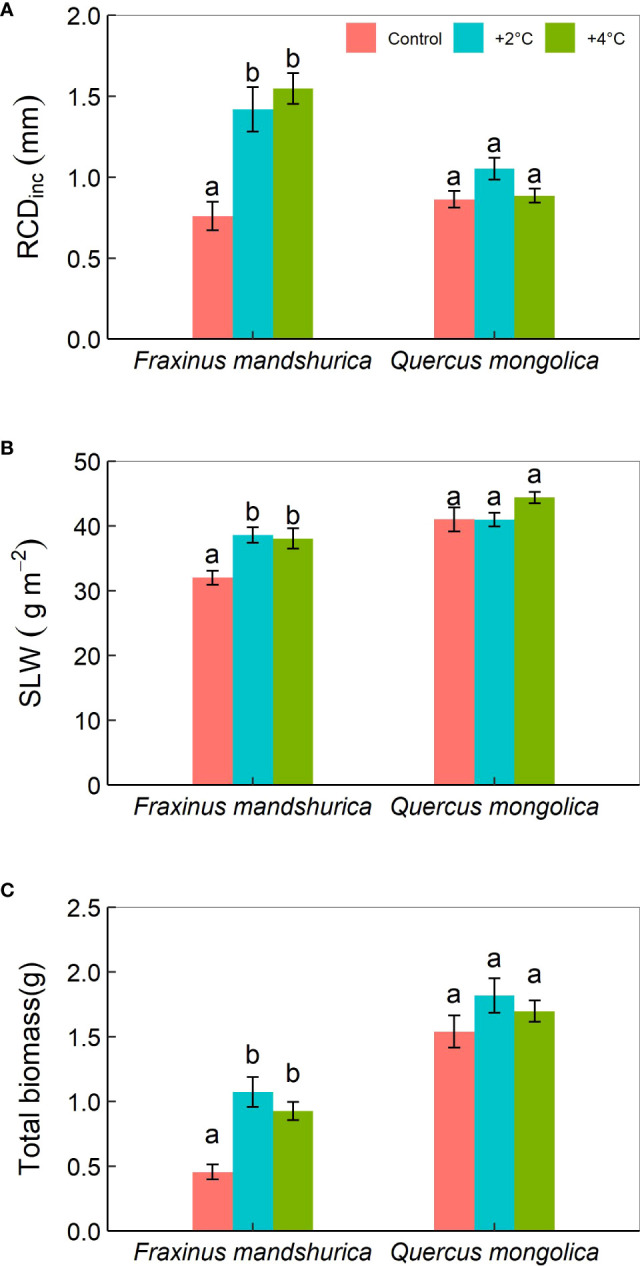
The mean periodic increment in root collar diameter (RCD_inc_) **(A)** and specific leaf weight (SLW) **(B)**, and total biomass **(C)** for different species growing under the three temperatures (control; +2°C; +4°C). The data were presented as the mean ± S.E. Different lowercase letters indicated significant differences (*P*<0.05) between temperature treatments for the same species.

### Seedling growth and biomass allocation pattern

The mean periodic increment in root collar diameter (RCD_inc_) was significantly affected by temperature, species, and their interactions. For *F. mandschurica*, RCD_inc_ under control temperature (0.76 mm) was greatly less than that under +2°C (1.42 mm) and +4°C (1.55 mm) treatments, while no difference was detected between these two increased temperature scenarios (**Figure 1A**). The mean periodic increment in total height (H_inc_) was little affected by species. There was a significant effect of temperature and species on specific leaf weight (SLW). SLW in the control temperature was lower than in the +2°C and +4°C treatments (**Figure 1B**). Total biomass was determined by temperature and species, but unaffected by precipitation levels and any interactions among the three factors. Seedlings at +2°C treatment had maximum final biomass (1.5 g) (**Figure 1C**).

**Figure 2:**
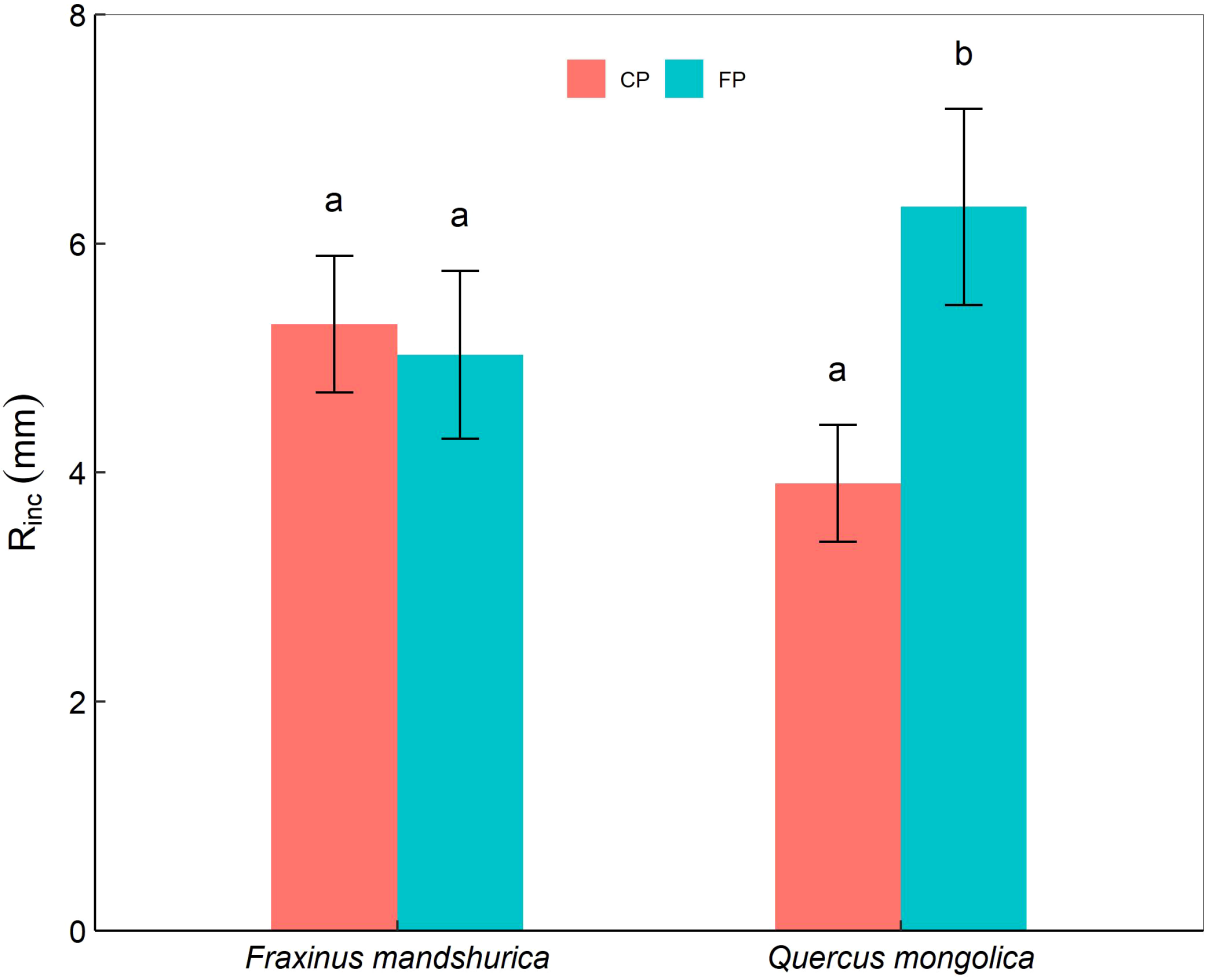
The mean periodic increment in maximum root length (R_inc_) of *Quercus mongolica* and *Fraxinus mandschurica* seedlings across the species and irrigating (current precipitation, CP; future precipitation, FP) treatments. The data were presented as the mean ± S.E. Different lowercase letters indicated significant differences (*P*<0.05) between precipitation treatments in the same species.

Furthermore, there were significant interactions of species × temperature and species × precipitation on the mean periodic increment in root length (R_inc_). For *Q. mongolica*, seedlings in FP showed an obvious increase in root length than those in CP (**Figure 2**). In addition, *F. mandschurica* seedlings in +2°C and +4°C treatments had a longer root length than those in the control (**Table 2**).

**Table 2:** Summary of statistics (F and P values).

	F			*P*	F	*P*	F	*P*	F	*P*	F	*P*	F	*P*
	RCD_inc_				H_inc_		R_inc_		Final biomass		Root- shoot ratio		SLW	
Temperature	13.67	**0.00**			0.29	0.75	2.24	0.11	7.31	**0.00**	27.70	**0.00**	4.66	**0.01**
Precipitation	0.01	0.94			0.00	0.95	2.30	0.13	1.31	0.26	0.09	0.77	1.08	0.30
Species	21.52	**0.00**			3.82	**0.05**	0.26	0.61	101.55	**<0.0001**	293.84	**<0.0001**	27.93	**0.00**
T×P	0.54	0.59			1.31	0.27	0.34	0.71	0.12	0.89	1.79	0.17	0.23	0.79
T×S	12.39	**0.00**			0.30	0.74	5.01	**0.01**	1.52	0.22	2.94	0.06	2.37	0.10
P×S	0.35	0.56			0.75	0.39	4.87	**0.03**	0.10	0.75	0.16	0.69	2.33	0.13
S×T×P	0.66	0.52			0.90	0.41	1.59	0.21	0.30	0.74	3.40	**0.04**	0.13	0.88
	Ψ				Fv/Fm		Total chlorophyll		Chlorophyll a:b		N concentration		C:N ratio	
Temperature	0.82		0.44		28.05	**0.00**	78.02	**<0.0001**	93.83	**<0.0001**	5.81	**0.01**	4.03	**0.03**
Precipitation	2.54		0.11		0.84	0.36	1.19	0.28	0.94	0.34	0.96	0.34	1.60	0.22
Species	47.85		**0.00**		42.47	**0.00**	9.59	**0.00**	1.40	0.24	3.42	0.08	12.13	**0.00**
T×P	4.32		**0.02**		0.16	0.85	1.14	0.33	1.32	0.27	1.05	0.37	1.23	0.31
T×S	1.82		0.17		0.39	0.68	12.29	**0.00**	7.54	**0.00**	3.62	**0.04**	2.69	0.09
P×S	0.23		0.63		0.78	0.38	0.92	0.34	0.39	0.53	3.10	0.09	3.74	0.07
S×T×P	0.04		0.96		0.14	0.87	0.72	0.49	0.84	0.44	1.53	0.24	2.36	0.12

Differences in all variables were tested by using factorial ANOVA (F values) across the different experimental factors (temperature, T; species, S; precipitation, P). Significant results were in bold. RCD_inc_, mean periodic increment of root collar diameter (RCD); H_inc_, mean periodic increment of height; R_inc_, mean periodic increment of maximum root length; SLW, specific leaf weight.

**Figure 3:**
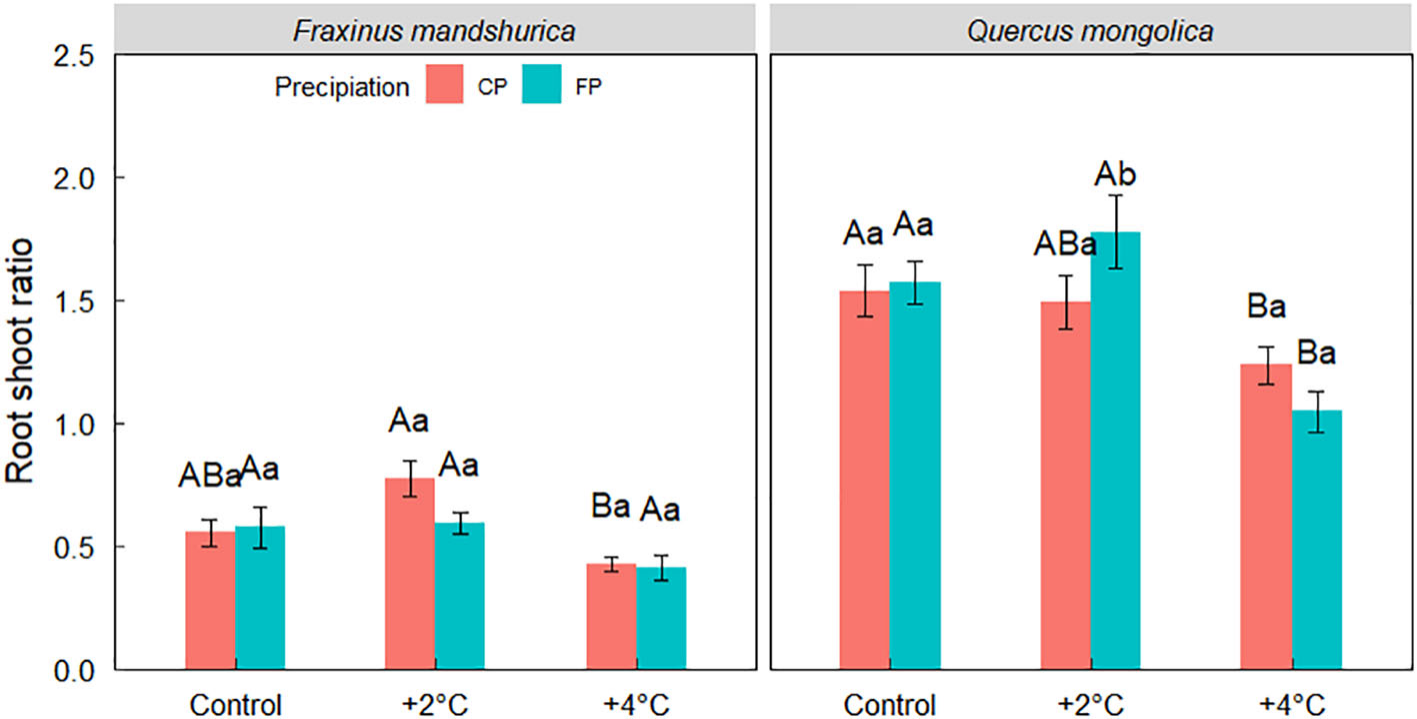
The root-shoot ratio of *Quercus mongolica* and *Fraxinus mandschurica* seedlings across the temperature (control; +2°C; +4°C) and irrigating (current precipitation, CP; future precipitation, FP) treatments. The data were presented as the mean ± S.E. Different capital letters indicated significant differences (*P*<0.05) among temperature treatments. Different lowercase letters indicated significant differences (*P*<0.05) between precipitation treatments in the same temperature condition and species.

However, a significant interaction among three factors was detected in the root-shoot ratio. For *Q. mongolica* under +2°C, seedlings from FP exhibited a higher root-shoot ratio than from CP, but no significant variance in the root-shoot ratio was observed for *F. mandschurica* (**Figure 3**).

### Physiological variables of seedlings

**Figure 4:**
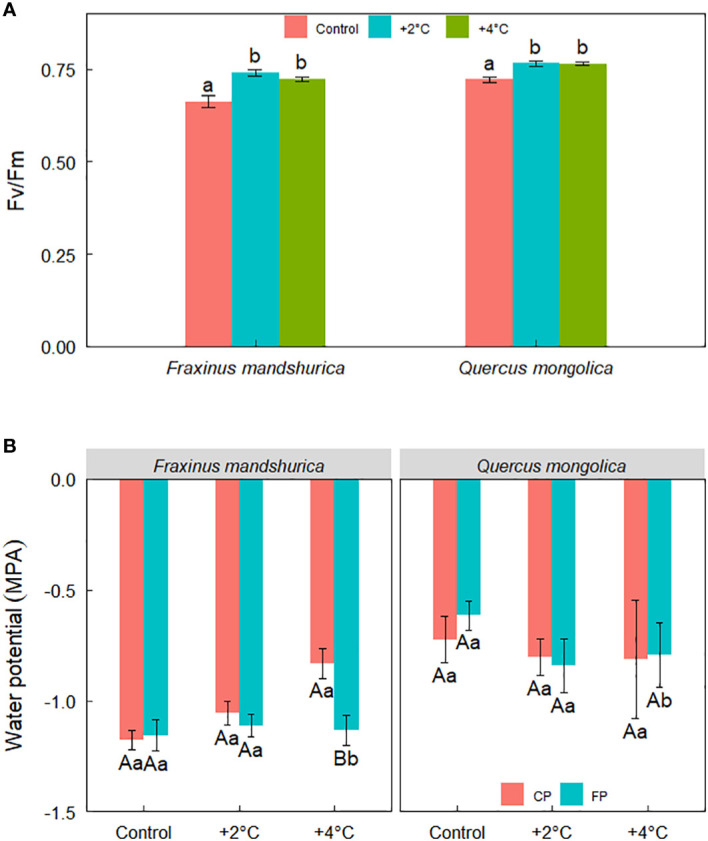
Fv/Fm for different species growing under the three temperatures (control; +2°C; +4°C) **(A)** and water potential across temperatures, precipitation treatments, and species **(B)**. The data were presented as the mean ± S.E. Different lowercase letters indicated significant differences (*P*<0.05) between temperature treatments in the same species for Fv/Fm. Different lowercase letters indicated significant differences (*P*<0.05) between precipitation treatments at the same species and temperature level while different capital letters indicated significant differences (*P*<0.05) between temperature treatments in the same species and precipitation level for water potential.

Fv/Fm was significantly affected by temperature treatment and species. Overall, plants from the control temperature performed a lower Fv/Fm than the rest two temperature treatments (**Figure 4A**). In addition, there was a significant interaction between temperature and precipitation treatment on the water potential of seedlings. Under +4°C, both species from FP showed a lower water potential than seedlings from CP (**Figure 4B**).

**Figure 5:**
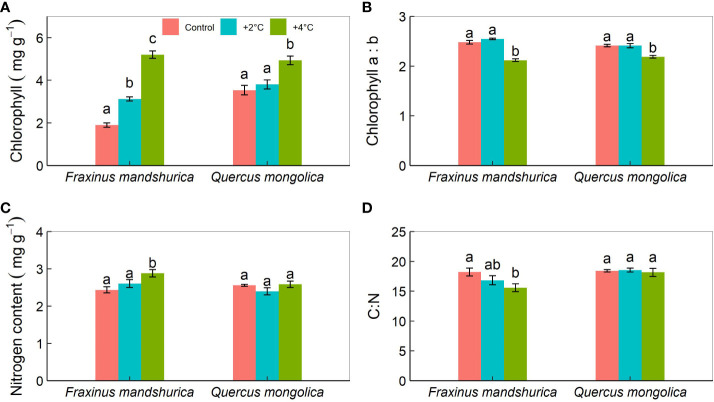
Total chlorophyll content **(A)**, the ratio of chlorophyll a:b **(B)**, nitrogen content **(C)**, and the ratio of C:N **(D)** in leaves for different species growing under the three temperatures (control; +2°C; +4°C). The data were presented as the mean ± S.E. Different lowercase letters indicated significant differences (*P*<0.05) between temperature treatments for the same species.

Total chlorophyll content was affected by temperature levels, species, and their interaction. For *Q. mongolica*, seedlings at +4°C treatment had a higher chlorophyll content than those at +2°C and control temperatures (**Table 2**). For *F. mandschurica*, significant differences in any temperature treatment on chlorophyll contents were observed, following the order: +4°C>+2°C>control temperature (**Figure 5A**). The ratio of chlorophyll a and chlorophyll b was affected by temperature levels and the interactions between temperature and species, showing that seedlings in +4°C had a lower ratio of chlorophyll a and chlorophyll b than those in +2°C and control treatments for two species (**Figure 5B**).

Foliar nitrogen concentration differed between temperatures and temperature × species interaction. For *F. mandschurica*, nitrogen concentration under +4°C treatment was greatly more than that under +2°C and control treatments (**Figure 5C**). C:N differed among temperature treatments and species (**Figure 5D**). Compared to the control treatment, C:N ratio of seedlings at the +4°C treatment significantly decreased by ~7.8% (**Table 2**).

## Discussion

In this research, we evaluated the effects of warming and decreased precipitation on the seedling performance of *Q. mongolica* and *F. mandschurica*. Temperature manipulations increased root collar diameter, specific leaf weight, total biomass, chlorophyll contents, nitrogen concentration, and Fv/Fm, but reduced the ratio of chlorophyll a/b and the C:N ratio. In addition, temperature and precipitation had no pronounced effects on seedlings’ height. Decreased precipitation only induced longer roots. When +2°C and precipitation acted concomitantly, an increased root-shoot ratio was observed. A temperature of +4°C and reduced precipitation induced more negative water potential. The two species did not respond to climatic manipulations in the same manner, with *F. mandschurica* responding more to warming and *Q. mongolica* responding to warming plus precipitation reduction. The different responses of the two species to climate change could provide us with more information about community dynamics in the future.

### The effect of climatic change on seedling growth

The root collar diameter and height are the most common indicators in regeneration research (Nissinen et al., 2020). It is widely reported that moderate warming can stimulate plant growth (Noh et al., 2021). In our study, warming (both +2°C and +4°C) increased the root collar diameter of the seedlings (**Figure 1A**). Similar results were obtained by (Fisichelli et al., 2014; Han et al., 2015), who found a 3 °C increase in temperature promoted an increase in root collar diameter in *Pinus koraiensis* Siebold et Zuccarini and *Abies holophylla* Maxim, by 10% and 7%, respectively. The growth of root collar diameter is derived from the activity of cambium, which begins with the increase of seedling height in spring and continues to grow even after the seedling stops growing (Yun et al., 2016). The height growth is related to a stem originating from the previous bud, which starts with bud sprouting in spring and stops with budburst in early spring (Kramer and Kozlowski, 1979). According to our results, warming had a positive effect on the root collar diameter of the seedlings while there was no obvious difference detected in seedling height among temperature treatments. The difference in the origination of root collar diameter and height might result in their inconsistent response to warming. We cultivated seedlings from June when the stem had stopped growing. Therefore, a response in seedling height would be expected to early spring and long-term warming. For example, the total heights of Silver birch (*Betula pendula* Roth.) and Scots pine (*Pinus sylvestris* L.), seedlings under the warming treatment were greater than under ambient environments after two growing seasons, while there were no differences observed during the first growing season (Nissinen et al., 2020). These outcomes speculate that seedling height does not immediately respond to climate alterations, at least in the short term. Height and root collar diameter do not represent the seedling growth performance due to lateral extension growth (Lu et al., 2018). Since the carbon storage of plants could be characterized by biomass, which is a better indicator of growth variations to environmental change. *F. mandschurica* had higher biomass under the +2°C and +4°C treatments than the control. The results were supported by (Prieto et al., 2009), who showed that warming increased the growth and total biomass of *Pinus halepensis* L. Increased biomass with warming might be owing to photosynthesis enhancement in the study areas (Slot et al., 2018). Our results imply that biomass was sensitive to climate variations (e.g., temperature). We hypothesized that drought-tolerant species like *Q. mongolica* could be more adapted to future climate changes than *F. mandschurica* (hypothesis 2). Our findings demonstrate the increase in root collar and biomass was consistent with the opinion that warming could promote growth but disagree with hypothesis 2, that there would be a greater growth increase in *F. mandschurica* compared to *Q. mongolica.*

Trees could enhance resource efficiency by adjusting portioning to different organs of biomass to adapt to conditions (Chmura et al., 2017). However, the response of specific species to changed environmental conditions is still unclear. Warming and precipitation reduction might have a contrasting effect on the biomass allocation pattern, depending on the trade-off mechanism for the adaption to temperature and the acquisition of water. In our case, *Q. mongolica* under the +4 °C condition had a lower root-shoot ratio than under +2°C and control conditions, and the same trend was found in *F. mandschurica*, which indicates that +4°C improves above-ground carbon storage for the two species. Moreover, combined with seedlings’ growth, warming might promote seedlings’ growth by increasing the allocation of carbon aboveground (Noh et al., 2020). Under +2°C, *Q. mongolica* from FP had a higher root-shoot ratio than seedlings from CP. This increase in the proportion of biomass allocated to the root could enhance the resistance of *Q. mongolica* to drought and induce more imbalance in water ecology (Grossnickle, 2012). Furthermore, this demonstrates that *Q. mongolica* is more sensitive to decreased soil moisture than *F. mandschurica*. In addition, root length is a paramount trait that is required to obtain water and nutrient resources in deeper soil layers (Kuster et al., 2012). The longer root of *Q. mongolica* with future precipitation alterations indicates that the root performance of *Q. mongolica* is consistent with the distribution of soil moisture. However, warming only affected the root length of *F. mandschurica.* Therefore, our results do not support that warming alleviates the effects of precipitation reduction (hypothesis 1). Numerous studies confirmed that oaks are drought-tolerant species with expanding their distribution in drier environments (Hu et al., 2013), and our results support that drought-tolerant species like *Q. mongolica* would expand their competitive advantage in future arid environments rather than warming environments (hypothesis 2).

Leaf traits and particularly specific leaf weight (SLW) are considered highly informative (Scheepens et al., 2010), as SLW reflects the capacity of plants’ carbon assimilation. In general, SLW increases with greater photosynthesis and carbon accumulation (Grassi et al., 2001). For *F. mandschurica*, the seedlings exhibited a greater SLW in the +4°C environment than in the +2°C and control environments. Previous studies showed that warmer environments enhance photosynthesis and net primary productivity (Rustad et al., 2001; Wu et al., 2011), and our results supported the above view through the SLW change. Through the seedlings’ growth performance, we overturn hypothesis 2 because *F. mandschurica* grew better than *Q. mongolica* in warming treatments.

### Climatic change effect on seedling physiology

The Fv/Fm ratio is considered to show damage to the PSII reaction center and is a good indicator of the conversion efficiency of intrinsic energy in the PSII reaction center (Ismail et al., 2014). Fv/Fm showed a trend where seedlings from the two species in the +4°C and +2°C environments had higher values than the seedlings in the control environment. This indicates that warming might promote the activity of the PSII reaction center. In addition, the two warming treatments enhanced total chlorophyll content for both species, but only +4°C had an obvious increase compared to the control for *Q. mongolica*. The elevated temperature often promotes the concentration of pigments, such as chlorophyll and other chemical tissues (An et al., 2017), thus we speculate that an increase of 4°C and 2°C might provide a near-optimal temperature range for chlorophyll synthesis and enhance the efficiency usage of radiation energy. *Q. mongolica* grows in warmer environments, such as ridges and sunny slopes, than *F. mandschurica*, thus *Q. mongolica* needs a higher temperature to synthesize chlorophyll than *F. mandschurica*. The increase in activity of the PSII reaction center and pigments might be two important reasons for the upregulation of photosynthesis under elevated temperatures during the growing season (Song et al., 2019). In our study, the chlorophyll a/b ratio decreased with temperature, indicating that chlorophyll b is more sensitive to warming than chlorophyll a. The chlorophyll a/b ratio reflects plants acclimatized to low-light environments (Wang et al., 2021). Therefore, in our study, the chlorophyll a/b ratio diminished with temperature suggesting that seedlings might not adapt to low light conditions in the warmer future. FP resulted in more negative water potential than CP for seedlings under the +4°C condition. This result indicates that the interaction of +4°C and precipitation reduction induces harsh conditions. Reduced precipitation provoked a decrease in water potential and an increase in root length but had no impact on seedling growth, which suggests that reduced precipitation does not limit the growth of seedlings for the two species. This phenomenon might result from the fact that the study site is in a humid region and decreased precipitation does not provoke a large reduction in soil moisture to limit growth.

The experimental warming reduced the foliar C/N ratio, and seedlings under the +4°C condition had a lower C/N ratio than under the control condition, which was mainly induced by a foliar nitrogen concentration increase, especially in *F. mandschurica*. Warming promotes soil nitrogen mineralization, thus increasing foliar nitrogen content (Xu et al., 2012). In Harvard forests, a 5 °C increase in soil temperature provided more available nitrogen for plants (Melillo et al., 2002). In addition, the biogeochemical hypothesis indicates that the increase of foliar N concentrate under elevated temperature is correlated to strengthened plant photosynthesis and increased plant nitrogen absorption capacity of (Zhang and Wang, 2021). The above two pathways could explain higher foliar nitrogen in a warming scenario. Further, 75% of foliar N concentrate is used for synthetic chlorophyll, which is supported by our results with higher chlorophyll contents under warming scenarios. Furthermore, a foliar N increase also confirmed an increase in the seedlings’ growth and was associated with increases in foliar photosynthesis activity. The seedling’s physiological performance in foliar N, Fv/Fm, and pigments are consistent with the fact that that warming promotes seedling growth. Furthermore, the physiological performances of *F. mandschurica* are better than *Q. mongolica* under the warming context, which suggests that *F. mandschurica* is more adapted to warming environments. Therefore, our results disagreed with hypothesis 2.

## Conclusions

Evaluating the responses of coexisting species with contrasting functional traits to projected climate changes is paramount in predicting the regeneration patterns of tree species and community dynamics. Our results indicated that interspecific differences largely decided the seedlings’ performance in future climatic conditions. *F. mandschurica* (a drought-intolerant species) seemed to be less sensitive to precipitation reduction, but have higher plasticity (e.g., SLW and foliar nitrogen content) in adapting to warming. A drought-tolerant species such as *Q. mongolica* actively accommodated precipitation reduction by increasing root length and inducing more negative water potential. In addition, warming promoted the seedling growth of the two species while reduced precipitation and warming with precipitation reduction merely induced traits related to the water balance in *Q. mongolica.* In the future, drought-tolerant species (e.g., *Q. mongolica*) might expand their range to drier (warming and precipitation co-occurrence) regions whereas *F. mandschurica* is better adaptative to warming environments. However, enclosed environments might underestimate the impacts of reduced precipitation and warming, and similar studies should be conducted in the field.

## Data availability statement

The raw data supporting the conclusions of this article will be made available by the authors, without undue reservation.

## Author contributions

QY conceived the ideas and designed the study. JY, JW, JX, and RL collected field data. JY analyzed the data and led the writing of first draft of manuscript. QY and JY substantially contributed to revising the manuscript. All authors contributed critically to the draft and gave final approval for publication.

## Funding

This research was funded by the National Natural Science Foundation of China (U1808201), and the National Key R & D Program of China (2020YFA0608100).

## Acknowledgments

We thank Prof. Jiaojun Zhu for his suggestions on the design of the study. We thank Miss Ting Zhang and Xinlei Yu for their help during the sample measurements. We thank Dr. Deliang Lu and Lining Song for their suggestions on the statistical analysis.

## Conflict of interest

The authors declare that the research was conducted in the absence of any commercial or financial relationships that could be construed as a potential conflict of interest.

## Publisher’s note

All claims expressed in this article are solely those of the authors and do not necessarily represent those of their affiliated organizations, or those of the publisher, the editors and the reviewers. Any product that may be evaluated in this article, or claim that may be made by its manufacturer, is not guaranteed or endorsed by the publisher.

## Abbreviations

SLW, specific leaf weight; RCD_inc,_ mean periodic increment of root collar diameter; R_inc_, mean periodic increment of maximum root length; H_inc_, mean periodic increment of height; Ψ, water potential; CP, current precipitation; FP, future precipitation.
