# Supplementary material for: Different responses of growth and physiology to warming and reduced precipitation of two co-existing seedlings in a temperate secondary forest
Source: Front Plant Sci. 2022 Oct 14;13:946141. doi: 10.3389/fpls.2022.946141 (PMC9614434; doi:10.3389/fpls.2022.946141)

**Supplementary Material:**

[Figure 1](javascript:;). Soil moisture in pots for planted *Quercus mongolica* and *Fraxinus mandschurica* seedlings across the temperature (control; +2 ℃; +4 ℃) and irrigating (current precipitation, CP; future precipitation, FP) treatments from June to September. The data were presented as the mean ± S.E. Different capital letters indicated significant differences (*P*<0.05) among temperature treatments in the same precipitation condition and species. Different lowercase letters indicated significant differences (*P*<0.05) between precipitation treatments in the same temperature condition and species.


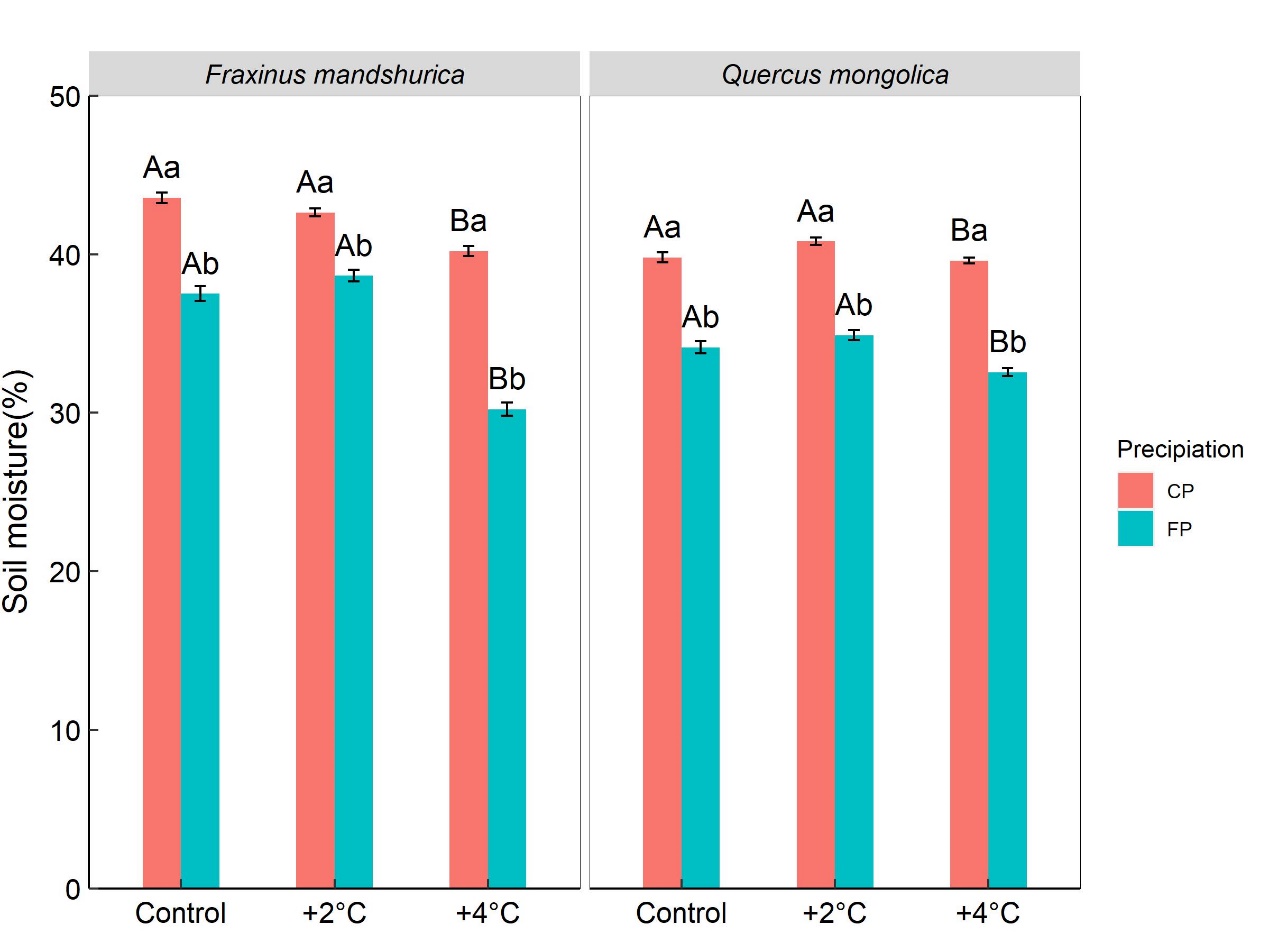

Supplement: Supplementary file 1 [file DataSheet_1.docx]
